# The Impact of Activity-Based Interventions on Neuropathic Pain in Experimental Spinal Cord Injury

**DOI:** 10.3390/cells11193087

**Published:** 2022-09-30

**Authors:** Jing Chen, Norbert Weidner, Radhika Puttagunta

**Affiliations:** 1 Laboratory of Experimental Neuroregeneration, Spinal Cord Injury Center, Heidelberg University Hospital, 69118 Heidelberg, Germany; 2 Spinal Cord Injury Center, Heidelberg University Hospital, 69118 Heidelberg, Germany

**Keywords:** neuropathic pain, spinal cord injury, preclinical rodent models, activity-based interventions, allodynia, hyperalgesia, cognitive perception of pain, sensorimotor activation, inflammatory modulation, spinal cord neural circuitry remodeling

## Abstract

Physical activity-based rehabilitative interventions represent the main treatment concept for people suffering from spinal cord injury (SCI). The role such interventions play in the relief of neuropathic pain (NP) states is emerging, along with underlying mechanisms resulting in SCI-induced NP (SCI-NP). Animal models have been used to investigate the benefits of activity-based interventions (ABI), such as treadmill training, wheel running, walking, swimming, and bipedal standing. These activity-based paradigms have been shown to modulate inflammatory-related alterations as well as induce functional and structural changes in the spinal cord gray matter circuitry correlated with pain behaviors. Thus far, the research available provides an incomplete picture of the cellular and molecular pathways involved in this beneficial effect. Continued research is essential for understanding how such interventions benefit SCI patients suffering from NP and allow the development of individualized rehabilitative therapies. This article reviews preclinical studies on this specific topic, goes over mechanisms involved in SCI-NP in relation to ABI, and then discusses the effectiveness of different activity-based paradigms as they relate to different forms, intensity, initiation times, and duration of ABI. This article also summarizes the mechanisms of respective interventions to ameliorate NP after SCI and provides suggestions for future research directions.

## 1. Spinal Cord Injury-Induced Neuropathic Pain

Neuropathic pain (NP) represents a frequent secondary condition after spinal cord injury (SCI), manifesting primarily with sensory, motor, and autonomic dysfunction [1,2]. Epidemiological data show the estimated total pain incidence ranges from 60–80% in SCI patients [3,4,5,6]. Of these, a recent systematic review and meta-analysis described a high prevalence of NP (53%, or >50%) in SCI patients [7]. SCI-NP is characterized by spontaneous or stimulus-evoked pain and may be associated with dysesthesia and paresthesia [2,8,9]. Stimulus-evoked pain can be subdivided into allodynia (pain in response to normally non-noxious stimuli) and hyperalgesia (an increased sensation of pain in response to normally painful stimuli) [6,8]. Up to 80% of NP that develops within the first year after SCI tends to become chronic [9]. SCI-NP is one of the major concerns for patients, accompanying functional deficits, influencing rehabilitation, and further reducing a patient’s quality of life.

SCI-NP symptoms appear either at or below the level of SCI [10]. At-level NP occurs in a segmental distribution within the dermatome of the neurological level of injury and/or within the three dermatomes below the neurological level of injury but not extending beyond this; below-level NP is located more than three dermatomal levels caudal to the neurological level of injury [11]. Early research has endeavored to provide some insights into the possible underlying pathophysiological mechanisms involved in SCI-NP to approach this refractory issue, but they are still not fully understood. Initial research focused on SCI-NP being derived directly at the site of injury; however, emerging evidence revealed that alterations have been observed remotely from the injury site still within the spinal cord and/or the peripheral nervous system (PNS) [6]. After SCI, a range of peripheral and central nervous system (CNS) processes can contribute to prolonged pain and altered sensation. For example, a primary lesion to the spinal cord can lead to a state of hyperexcitability of the sensory nerve fibers in the PNS in response to external stimuli, a common pain phenomenon termed peripheral sensitization. In conjunction, central neurons in the spinal cord gray matter innervated by such sensitized nociceptors undergo dramatic functional alterations (hyperexcitability) termed central sensitization, which is also found in other forms of chronic pain [6,7,12]. In general, at-level NP is hypothesized to result from local damage to spinal nerve roots in addition to the spinal cord itself, typically developing in the subacute phase following SCI, due to peripheral and central sensitization phenomena [13]. However, below-level NP is typically not due to direct PNS damage at the location of pain presentation [10,11,14]. For example, changes in the processing of sensory input in below-injury spinal cord gray matter may be linked to pain-related signaling from the lesion site and maladaptive sensory processing in the distal spinal cord neural circuitry, and these remain persistent [6,8,10,11,13,14,15,16,17,18,19,20,21,22,23,24,25,26,27,28].

Current treatment strategies such as pharmacological and neurosurgical methods have shown partial benefits in SCI-NP mitigation. Pharmacological therapies such as antiepileptic drugs usually act by both strengthening inhibitory mechanisms and/or decreasing excitation in various pain etiologies [29]. However, most drugs can only provide around a third of patients with a 50% reduction in SCI-NP at best [6,29]. Common neurosurgical procedures for pain can be effective at relieving SCI-NP, for instance, a dorsal rhizotomy—lesion of the dorsal root entry zone (DREZ)—may reduce hyperactive input into dorsal horn neurons close to the level of injury, providing a relatively good (>50%) relief of at-level NP. In contrast, direct destructive surgical interventions in the spinal cord aiming to interrupt transmission of NP-related signals such as cordotomy or cordectomy did not result in relevant pain relief [6,30].

SCI-induced paresis or plegia results in altered if not absent motor output and, as a consequence, significantly diminishes sensory input [31,32]. Various types of activity-based interventions (ABI, e.g., treadmill training, wheel running, walking, swimming, and bipedal standing as well as cycling, shallow water walking, etc., however, the latter were not investigated in relation to SCI-NP) engaging paretic limbs have been used to prevent/reverse NP in rodents following experimental SCI. Although not applicable to the most severe SCI patients, ABI represents a non-invasive treatment, which may be better tolerated than some invasive treatment options and can be individually modified based upon a patient’s recovery. It is critical to clarify the current understanding of mechanisms underlying such interventions in alleviating chronic pain to assist researchers in identifying the gaps in knowledge, which can then be properly addressed for the further refinement of respective strategies.

The main goals of this review are to (1) review some of the known mechanisms underlying NP after SCI, (2) categorize and identify ABI-related parameters that modulate these underlying mechanisms, and (3) finally to provide suggestions for future studies. The focus of this review is on molecular and cellular mechanisms by which ABI alleviates NP behavior in SCI rodent models. In the literature, a variety of terms are used to describe ABI, e.g., training, exercise, physical exercise, and sensorimotor activation. To improve the consistency of terminology in this review, all respective interventions aiming for the activation of paretic limbs will be termed ABI. The type of intervention in each study will then be specified.

## 2. Included Rodent Models of SCI-NP

Based upon the principal purpose of the present work, we strictly performed search procedures with the following inclusion and exclusion criteria. In PubMed, the search phrases “spinal cord injury,” “neuropathic pain or allodynia or hyperalgesia,” and “exercise or training or activity-based interventions” were combined using the “AND “ operator, and the query was (“spinal cord injury”) AND ((neuropathic pain) OR (allodynia) OR (hyperalgesia)) AND ((exercise) OR (training) OR (activity-based interventions)). Only those preclinical studies containing all three search components with “exercise” or “training” or “activity-based interventions” as the primary treatment for SCI-NP were evaluated in this review. According to this principle, the screening after the initial search turned up 23 studies, we excluded those in which the lesion did not result in the development of NP symptoms, behavioral tests unrelated to NP, or the ABI paradigm started already prior to SCI [33,34,35,36]. As a result, this review features the remaining 19 studies. Here, we then list relevant animal studies on the topic (Table 1) and discuss the given characteristics to provide direction for future studies.

### 2.1. Characteristics of Included Experimental Models

Of the research discussed here, rats account for 79% (15 of 19 studies) of the total, and mice contribute 21% (4 of 19 studies). Similar to human SCI, rodent experimental SCI is characterized by pathophysiological changes. In general, rats tend to be less anxious and better suited for behavioral analysis than mice [41,42,43]. On the other hand, modern genetic tools with transgenic and knockout lines in mice have increased the utilization of the mouse model [44]. However, it should be taken into account that different strains of mice, such as BALB/c and C57BL/6, have varying innate immune and behavioral responses to stress, which could account for observed differences in threshold sensitivity to innocuous stimulation [45,46,47].

Only 32% (6 of 19 studies) of the research described here used a male rodent model of SCI, while the studies have been predominantly (~68%, 13 of 19 studies) conducted on young female rodents. This may be attributed to the fact that female animals have shorter urethras than their male counterparts; this anatomical sex difference makes it easier for researchers to fully empty female urinary bladders and lowers the risk of urinary tract infection following SCI [42,48]. Additionally, females are more docile and less aggressive, making them better candidates for group housing following surgeries, which leads to better outcomes compared to solitary housing. However, in the clinic, approximately 70% of SCI patients are male [49]. Furthermore, it has recently been demonstrated in SCI-NP models that sometimes male and female animals interpret nociceptive stimuli differently and sometimes they do not, indicating a lack of understanding on whether sex plays a role in SCI-NP and what mechanisms underly such differences [50,51,52,53]. There is a need for further exploration into sex differences contributing to SCI-NP and the mechanistic pathways involved.

Given the variety of SCI lesion types and severity in patients with NP, animal models used in this field try to replicate this range as much as possible. Among the included studies, the moderate contusion and compression of mid-to-low thoracic (T6-T13) were the most commonly used SCI-NP models (79%, 15 of 19 studies). The primary purpose of using these injury models was to simulate the development of human incomplete SCI while producing a stable and consistent NP state that allows for mechanistic examination [42]. The majority of these moderately injured animals were then able to regain weight support function, which was an essential criterion for some of the following weight-bearing interventions and NP-related behavioral assessments. Once SCI-NP develops in these models it remains chronic, allowing for early and delayed ABI. In addition, at-level NP is a frequent sequel of cervical SCI in humans. Therefore, an injury model mimicking this type of injury is relevant. Three studies performed a cervical (C5) hemicontusion SCI on rats allowing for the examination of NP in forelimb dermatomes that were innervated by the injured neurological levels [17,20,24]. The relatively moderate degree of the animals’ injury severity allows wheel running to begin as early as 5 days after SCI [15,22]. One study used rat SCI models of C6 posterior or lateral compression to successfully induce NP-related symptoms, such as mechanical and thermal sensitivity and supraspinal pain [28]. This specific injury aimed to mimic spinal cord compression injuries in patients, which can result from the ossification of the posterior longitudinal ligament, cervical spondylotic myelopathy, and spinal cord tumors. The persistent SCI-NP-related behavioral performance and gradual motor functional recovery of animals allow the use and examination of continuous ABI. Due to the necessity of weight-support for interventions and assessments, the range of severity of SCI-NP models tends to be limited and new methods should be considered to simulate the SCI-NP experienced by patients with more complete SCI.

### 2.2. SCI-NP Measurements of Experimental Models

SCI-NP evaluation in current preclinical studies (Table 1) primarily focuses on identifying stimulus-evoked pain in response to mechanical and heat stimulations by measuring stimulus-evoked behavior parameters (threshold/response rate/duration) [54]. Among the selected studies, withdrawals in response to a non-noxious mechanical stimulus (95%, 18 of 19 studies, using von Frey filaments) or a noxious heat stimulus (74%, 14 of 19 studies, using Ugo Basile Plantar Heat Test) were used as readouts; only 4 of 19 studies (21%) reported the outcome of the response to cold allodynia (using Cold Hot Plate Test). In addition, most of these assessments were applied to the hindpaws of the animals to examine below-level pain after a thoracic or cervical moderate contusion/compression that allows partial recovery of motor function to regain weight support. Only five studies assessed at-level NP outcomes [17,20,24,52,53], two of which implicated the testing on the trunk area, and the other three examined the withdrawal response of the forepaws. The infrequency can be attributed to the complexity of implementing at-level NP testing on the trunk area first with a brush for a graded response which was then switched to a mechanical probe method (such as a von Frey hair) or pressure test applied by a pinch of a greater skin region, again with a graded response to the stimulus. Further, it is difficult to detect where exactly the probe touched the fur-covered skin area or what response was elicited. Moreover, when an object approaches above its head, an animal may become extremely anxious and try to escape [55]. Therefore, at-level NP assessments need further examination.

Even though rodent SCI models generally used in preclinical studies mimic the injury process and pain-evoked responses of the human CNS, pain also incorporates subjective factors such as spontaneous and emotional components. For example, clinically evaluating spontaneous pain extent incorporates subjective factors while using a questionnaire with SCI patients [56,57]. For obvious reasons, it is not possible to apply the same methods to rodents. Therefore, it is becoming increasingly important that preclinical NP measurements incorporate the evaluation of spontaneous continuous pain and/or emotional components to provide improved clinical applications. The closest associated behavioral test for this would be the grimace scale used to measure characteristic facial expressions associated with spontaneous pain in rodents and other mammals [58]. However, the scales have not been widely adopted since they are time-consuming and may be challenging for inexperienced experimenters due to more subjective classifications [59,60,61]. The observation of spontaneous NP was performed only in one of the included studies, by recording the number of freezing episodes when animals had random activities in an open field [19]. However, some other SCI-related complications such as spasticity, numbness, motor dysfunction, etc., can also cause freezing actions [50], which could also impede the use of some operant tests and still needs to be clarified with further examination. Over the past several years, new testing paradigms were added for operant testing of cognitive perception of pain [22,24,25,29]. The common idea of these paradigms is that SCI-NP animals tend to escape and/or avoid an aversive stimulus. Animals have the active choice between a naturally preferred dark environment with stimuli that may induce pain or an aversive light area without stimuli. Operant tasks included the consideration of the emotional–affective component of SCI-NP, by evaluating whether a rodent is motivated to escape a painful stimulus (Place-Escape Avoidance Paradigm, PEAP, and the Mechanical Conflict-Avoidance System/Paradigm, MCAP) or spend more time in an environment previously associated with pain relief (Conditioned Place Preference, CPP). The utility of these operant tasks has incorporated a conscious element to provide a more complete view of SCI-NP outcomes in preclinical studies. However, very few published studies on this topic have incorporated operant tasks in their research, even though many of these studies are moderate incomplete SCI studies and are not ones that lack mobility (Table 1). Future validation research should take efforts toward integrating multi-dimensional NP assessments in available studies in order to more comprehensively interpret findings.

## 3. Mechanisms of SCI-Induced NP

Since the studies examined here have selectively investigated below-level NP mechanisms, the following part of this section will primarily speak about below-level NP-related maladaptive changes of the immune system and neurotrophic mediators as well as functional and structural changes in the spinal cord gray matter circuitry correlated with pain behavior providing a theoretical background for exploring the mechanisms by which ABI attenuates SCI-NP.

### 3.1. Maladaptive Changes of Immune System and Neurotrophic Mediators

When SCI occurs, reactive immune cells (e.g., macrophages) infiltrating the SCI lesion are derived from recruited monocytes from peripheral circulation or arise from activated resident microglia, which is typical in CNS neuroinflammatory regulation [62]. While at the same time, locally there is an immediate neuroglial activation in response to nerve damage (involving endothelial cells, microglia, and astrocytes) that initiate the dysfunction and maladaptive modulations of pro-and anti-inflammatory cytokines/mediators (e.g., pro-inflammatory cytokines: tumor necrosis factor–α (TNFα), interleukin 1β (IL1β), interleukin 6 (IL6); anti-inflammatory cytokines: interleukin 4 (IL4) and interleukin 10 (IL10)) and neurotrophic factors (e.g., brain-derived neurotrophic factor (BDNF), glial cell-line derived neurotrophic factor (GDNF), nerve growth factor (NGF), neurotrophin 3 (NT-3), etc.), that further contribute to driving maladaptive alterations within the CNS and induce subsequent impairments [63,64,65].

Pro-inflammatory cytokines such as TNFα, IL1β, and IL6 are thought to be released in response to neuroglial (microglial) activation following nerve damage [25,27,63,65]. Both TNFα and IL1β have been described to be markedly elevated at- and below-level of SCI and within the cerebrospinal fluid, suggesting SCI-induced inflammatory states in diverse CNS sections [25,27,65]. Previous research demonstrated a dramatically increased expression of TNFα and IL1β at- and below-level 12–15 weeks post-SCI, which was associated with the development of below-level thermal hyperalgesia and mechanical allodynia [23]. Further, these altered pro-inflammatory cytokines persisted up to 24 months post-SCI [25]. In contrast, the level of the anti-inflammatory mediators, IL4 and IL10, were unaffected at and below the level of injury, suggesting a lack of anti-inflammatory modulations in spinal circuits following primary injury. Together these findings suggest that a dysregulation of pro- and anti-inflammatory mediators may signal to distal regions from the SCI lesion site, leading to a persistent inflammatory state, which may further promote the formation and maintenance of below-level NP [54,64].

Previous evidence indicated that immune cell responses during early neuroinflammation can induce prolonged sensitization of central and peripheral processing of nociceptive stimuli, which may contribute to the formation of pain [24,62,66]. SCI-induced macrophage recruitment and activation may contribute to nociceptor sensitization by releasing several soluble mediators. For example, below-level mechanical allodynia was qualitatively associated with infiltrating ED1 expressing macrophages in the lumbar dorsal column and dorsal horn in a T13 rat lateral hemisection [66]. In contrast, a recent study demonstrated that similar numbers of macrophages are present bilaterally at a C5 hemicontusion lesion site and surrounding C7–C8 spinal cord levels (where forepaw somatosensory information is processed), regardless of SCI-NP occurrence, however, they were increased in SCI rats compared to naive controls [22]. Strikingly, the expression of ED1 expressing macrophages in at-level dorsal root ganglia (DRG) (not examined below-level) was strongly associated with the presence of at- and below-level NP behavior after SCI and persisted until 30 days post-injury (dpi) [22], indicating a role for DRG macrophage induction in mechanical allodynia. Unfortunately, the examination of macrophages in below-level DRG was not mentioned in this study and, therefore, still needs to be further explored. Following local microglial activation, a self-propagating mechanism of increased cytokine expression is initiated, triggering a cascade of inflammatory reactions in the CNS by producing cell-specific markers and releasing pro-inflammatory cytokines, reactive oxygen species, ATP, excitatory amino acids, and nitric oxide, among other things. Activated glia in the dorsal horn at multiple levels of the spinal cord enhances the release of neurotransmitters (in nociceptive modulatory circuits) and the excitability of second-order nociceptive (spinothalamic/projection) neurons, causing global pain alterations in the spinal cord during this period [64,67]. For instance, increased activation of Iba1-expressing microglial cells in the dorsal horn below the level of SCI is associated with the development and maintenance of below-level allodynia after SCI [24,29,67].

In the early stages of SCI, neurotrophic factors such as NGF, BDNF, and GDNF are released from activated microglia and are elevated following injury [67,68]. NGF and BDNF both play several roles in the development of mechanical allodynia, and GDNF is known to induce the expression of pain-relevant sodium channels [68]. On the other hand, in the subacute/chronic phase of SCI, some studies determined a loss of neurotrophic factors that have been associated with NP conditions in below-level SCI-NP [15,17,19,26]. The expression of BDNF in the distal spinal cord will gradually decrease, thereby possibly reducing the BDNF-dependent remodeling and repair of the spinal cord neural circuitry [24]. A decrease in BDNF and a reduced expression of NT-3 and synapsin I (a vesicle-associated phosphoprotein that is controlled by BDNF and NT-3) in the lumbar spinal cord was reported, with a consistent presence of mechanical allodynia and hyperalgesia [13]. The downregulation of BDNF and its high-affinity receptor tropomyosin-related kinase B (TrkB) was found within the lumbar spinal cord after a thoracic SCI, along with the drop of the core transcription factor CREB (that mediates gene transcription downstream of the BDNF/TrkB signaling) and p-CREB (that promotes the synthesis of glutamic acid decarboxylase-65/67, GAD-65/67), together are possibly responsible for reduced GABAergic inhibition and leading to the occurrence of below-level NP symptoms [24]. Furthermore, it was found that the reduced expression of the potassium-chloride cotransporter 2 (KCC2) in the distal spinal cord may be a consequence of the decreased BDNF-mediated TrkB activation, which suppresses the normal Cl-dependent fast inhibition in GABAergic neurons, therefore contributing to the formation of below-level NP [17]. The development of below-level NP also correlated with a marked reduction in the levels of GDNF and artemin (another one of the GDNF family of ligands, GFL) in the distal spinal cord and DRG. SCI-induced downregulation of GDNF and GFL was associated with a dramatic increase in the density and the distribution throughout the dorsal horn of GFL-responsive afferents (non-peptidergic isolectin B4 (IB4) expressing fibers) in rats with SCI-induced allodynia [15].

### 3.2. Spinal Cord Gray Matter Neural Circuitry Impairments

Nociceptive sensory information from the periphery is conveyed to the CNS (dorsal horn of the spinal cord), where it is processed and transmitted to the brain. After SCI, the inhibition of gray matter neuronal activity is disrupted [17,21,23,24,25], maladaptive plasticity in the superficial dorsal horn occurs [17,19,20,21,38], and firing from the nociceptive projection neuron increases [69].

SCI-NP is strongly associated with the hyperexcitability of the dorsal horn, which is thought to be driven by the shift of inhibitory interneurons [54] and the altered function of descending inhibitory and facilitatory pathways [70]. Emerging evidence has described additional pathophysiological mechanisms that result in decreased inhibition after SCI: (1) shift of GABAergic neuronal cells in the superficial dorsal horn of the spinal cord [23]; (2) downregulation of KCC2 in the superficial dorsal horn [19,25,27]; (3) reduction in GAD-65/67, which are enzymes responsible for the basal levels of GABA synthesis [21,24]. All of these alterations are responsible for the reduction of inhibitory synaptic transmission and enhanced excitatory input into spinal circuits.

After SCI, all aspects of sensory processing may be affected. Recent studies unearthed some of these changes in the spinal cord dorsal horn. Non-noxious stimuli are registered by Aβ-fibers that typically synapse in laminae III-V of the dorsal horn. Under normal conditions, superficial lamina I and II projection neurons are activated by noxious stimuli mediated by C- and Aδ-fibers. The peptidergic projections (CGRP+) terminate mostly in lamina I and outer lamina II (IIo), while the non-peptidergic projections terminates mostly to inner lamina II (IIi) [71]. However, following SCI, peptidergic C- and Aδ- calcitonin gene-related peptide (CGRP) expression increased within lamina I and II of the lumbar spinal cord accompanied by below-level NP symptoms [28]. Furthermore, CGRP-expressing fibers sprout into deeper laminae III and IV below the level of SCI, and the aberrant sprouting is strongly associated with the presence of below-level mechanical allodynia [17,18,19,20,21,38]. The sprouting peptidergic fibers could acquire access to circuits typically processing non-noxious mechanical stimuli and increased neuronal activity may contribute to central sensitization by raising the excitability of postsynaptic laminae III and IV neurons as well as the wide-dynamic range (WDR) neurons [72]. Indicating that this is new growth, aberrant CGRP-expressing fibers colocalize with growth-associated protein-43 (GAP-43) at spinal levels below the lesion associated with mechanical allodynia [38]. Additionally, non-peptidergic IB4-expressing C-fibers can sprout into laminae II and III following SCI, whereas under normal conditions IB4 binding was concentrated mainly in lamina IIi; this type of intraspinal sprouting of primary afferents seems to be related to the formation of below-level NP [15,20]. The downregulation of BDNF and GDNF expression below the level of SCI may be related to the aberrant sprouting of CGRP-expressing and IB4-expressing fibers, respectively [17,19,26]. However, conflicting findings from other studies have demonstrated that the aberrant sprouting of CGRP expressing fibers can be reversed after inhibiting BDNF signaling [38]. Therefore, clarifying research into the connections between neurotrophic factors, primary afferent changes, and SCI-NP is needed.

## 4. Preclinical Evidence of ABI in the Treatment of SCI-Induced NP

A growing body of research has focused on investigating the underlying mechanisms of ABI for alleviating SCI-NP. Researchers have used various SCI models (thoracic contusion, compression, cervical hemicontusion, and posterior/lateral compression) and intervention methods (treadmill training, wheel running, walking, swimming, and bipedal standing) to optimize ABI parameters (body weight support, intervention time point, ABI duration, intensity, and frequency) in the alleviation of NP (Table 1). This section categorizes ABI methods involved in existing research, highlighting the mechanisms upon which ABI modulates SCI-NP. Only the experimental groups in which the SCI animals were trained according to a specialized ABI protocol are listed in the tables, not the subgroups in which ABI was combined with pharmacological intervention or stem cell transplantation. The results in the tables are compared to those that were sedentary or untrained.

### 4.1. Early vs. Delayed Intervention

Preclinically, SCI-NP symptoms appear as early as 5–7 days after SCI and are stabilized by 2–4 weeks [13,14,15,16,17,18,19,20,21,22,23,24,25,26,28,33,34,35,36,37,38,39,40]. Therefore, early intervention was classified as ABI initiated within the first 13 dpi and delayed intervention for ABI initiated 14 dpi or later. This allows for the examination of the efficacy of interventions applied during the development or following the establishment of SCI-NP (Table 2).

The earliest ABIs following SCI were either voluntary (1 dpi walking in plexiglass balls, Clear Run-about Balls, [14]) or involuntary (4 dpi treadmill training with body weight support, 4 dpi swimming, and 4 dpi standing with body weight support [13]) and only partially effective. However, ABI initiated 5 dpi without body weight support (wheel running, and treadmill training) in several cervical and thoracic SCI models were found to alleviate NP [17,18,21,24,25,28,29] and even prevent mechanical allodynia from developing [15,22]. In general, early ABI provided SCI-NP relief; however, there were a couple of examples when either a relatively high amount of body weight support (75%) was utilized with treadmill training or voluntary walking in plexiglass balls introduced 8 dpi were found to increase below-level mechanical allodynia [14,38]. Likewise, when wheel running was introduced later at 14 or 28 dpi, it led to increased at-level mechanical allodynia [18]. However, on the whole, delayed interventions were successful in alleviating SCI-NP [18,22,25,27,53], except when body weight support was provided together with the reduced ABI intensity in comparison to their effective paradigms [40].

Two studies reported mice with moderate T9 contusion SCI, which underwent treadmill training by utilizing identical parameters for 4 weeks initiated in the early (7 dpi) or chronic SCI phase (42 dpi). The former study confirmed that these treatments appreciably alleviated mechanical and cold allodynia, and the latter one found a significant amelioration effect on mechanical allodynia and thermal hyperalgesia. Further, the interventions at both time points were able to alter aberrant peptidergic CGRP expressing nociceptive sprouting in the spinal cord deeper dorsal horn [19,20]. In rat midthoracic compression SCI, multiple treadmill training settings were investigated, and their findings demonstrated that either early or delayed ABI resulted in substantially reduced expression of pro-inflammatory cytokines TNFα and IL1β in diverse parts of the CNS, which is a sign of reduced inflammatory mechanisms [18,25,27].

Given that early and delayed interventions appear to be equally beneficial, studies that addressed how long the ABI needed to be applied for the benefit and if it lasted after it was removed were examined.

### 4.2. Long-Term vs. Short-Term ABI

A better understanding of how ABI duration and sustainability affect the outcome of pain therapy may aid in the development of more effective treatments. This section features successful short-term (≤4 weeks) and long-term (>4 weeks) ABI, with the majority of these treatments including five or more sessions per week. Interestingly, some of these studies examined the persistence of ABI efficacy after a few weeks of removal.

The research compiled in Table 2 reveals that either short-term or long-term ABI delivers the benefit of SCI-NP amelioration. The studies indicate that short (≤4 weeks) or long (>4 weeks) ABI duration does not matter, but the type of ABI and implementation are important. Short-term (2 weeks) plexiglass ball walking only provided partial NP alleviation, however, other short-term (1–4 weeks) instances of treadmill training (either with or without body weight support) and wheel running were able to prevent the onset of SCI-NP [15] or even reverse the NP states after its full expression [19,21,22,24,26,28,29]. The included studies show varied long-term ABI paradigms ranging from 7 weeks to 2 years. The 7-week intensive swimming and standing, both of which are ABI with body weight support, failed to fully mitigate NP [13]; in another example, animals were 80–90% supported of their body weight with robotic swing assistance for 8-week treadmill training resulting in ineffective NP amelioration [21]. Aside from these, the rest of the long-term ABI studies had a significant impact on the improvement of NP states [15,18,25,27,28,53].

The longest continuous ABI duration in the relevant studies was up to 2 years (without training intervals) [25], with results comparable to previous work utilizing the same thoracic moderate compression model with a 12-week ABI period [16,23]. Findings revealed that once inflammation develops, it persists through the chronic phase (12 weeks or 2 years) and was altered by ABI. This anti-nociceptive effect lasted, with pro-inflammatory cytokines levels in diverse parts of the CNS nearly restored to that of normal intact control animals. Then, during the 5-week ABI-off period following 12-week continuous ABI, they found that mechanical allodynia returned 1–2 weeks after removal, and thermal hypersensitivity increased 2–3 weeks after ABI ended, however, the anti-nociceptive effect on cold allodynia continued until the end of the entire study [16]. Following 12-weeks ABI when NP symptoms reappeared, alternations of anti- or pro-inflammatory cytokines levels were not examined [16]. This study suggests that ABI must be continued to be successful. On the other hand, there were also 4-week ABI-off paradigms in two studies, where it was discovered that the anti-nociceptive benefits lasted 4 weeks despite the removal of intervention after a 2-week or 8-week treadmill training with a body weight support period [17,21]. However, no mechanism-related alterations were examined after the cessation of ABI in these studies. Understanding why this reversal may or may not occur from a mechanistic level would be a natural progression of this work in the field.

As a result, although not conclusive, the overall trend appears to be that discontinuing ABI leads to the reversal of most analgesic effects; however, it is evident that whether ABI is implemented for short- or long-term, it is effective.

### 4.3. Full Weight-Bearing vs. Non-Full Weight-Bearing Rhythmic ABI

ABI may promote the remodeling of neural circuits after SCI through sensory feedback to reduce SCI-NP [24,73]. Rhythmic weight-bearing during ABI can promote activation of proprioceptive and mechanoreceptive afferents, which may drive attenuation of SCI-NP. The 19 included trials in Table 3 were classified into several types so as to identify key elements in successful interventions in the alleviation of SCI-NP. Full weight-bearing rhythmic ABI refers to intervention methods that require animals to carry their full body weight and alternate their limbs in a rhythmic pattern. In comparison to the other two groups, non-full weight-bearing rhythmic and full weight-bearing non-rhythmic ABI groups, full weight-bearing rhythmic ABI is the most beneficial for SCI-NP alleviation, likely due to the greatest activation of proprioceptive and mechanoreceptive sensory neurons.

Quadrupedal treadmill training, wheel running, and walking belong to the category of full weight-bearing rhythmic paradigms, and 77% (10 of 13 experiments) of interventions of this sort were successful in attenuating NP [15,16,19,20,22,23,25,26,28], except in instances when either plexiglass ball walking was started early (8 dpi) or wheel running was induced in the sub-chronic phase (14 or 28 dpi), which then increased both at- and below-level mechanical allodynia [14,18]. When applied at different timepoints (plexiglass ball walking started 1 dpi, and wheel running begun 5 dpi) they were found to be beneficial; therefore, it is likely to be due to the timing of such an intervention rather than the type of intervention strategy [14,15].

Non-full weight-bearing ABI such as treadmill training with body weight support and swimming seem to provide less effective NP alleviation, as only 44% (4 of 9) of studies showed a significant benefit [15,17,19,26], and 56% (5 of 9) of these were not able to fully improve NP states [15,23,52] and even raised the risk of below-level mechanical allodynia development [38]. These non-beneficial interventions may provide some insight. By comparing different treadmill training and body weight support settings in this category, it was observed that treadmill training paradigms that utilized a higher amount of body weight support (>75% of the animal’s body weight) and/or prolonged use of ankle swing assists, referred to as assisted stepping with passive movement of hindlimb joints, provided only partial NP relief and even increased NP [21,38]. Thus, sensory input engaged with active but not passive movement may serve as a key factor in this analgesic action. Therefore, active weight-bearing walking may be more helpful at relieving SCI-NP than passive bike training or robotic walking, which supports the majority of the patient’s body weight and aids joint movements. Furthermore, the greater the load from the animals’ body weight during ABI, the larger the beneficial effects.

A similar concept is supported by another study [13], standing with box support can be categorized as a non-full weight-bearing non-rhythmic ABI. The animals had a box to help carry their body weight without rhythmic limb movements during standing [13], which provided no benefit in decreasing mechanical allodynia. They also compared outcome measures after treadmill training with body weight support, standing, and swimming (non-full weight-bearing rhythmic ABI) to evaluate the possible contribution of sensory input related to hindlimb load and movement rhythmicity to nociceptive function. They discovered that rhythmic, weight-bearing treadmill training was the most effective at alleviating mechanical allodynia (standing had no impact and swimming had only a transitory effect) [13].

Two studies utilizing treadmill training and body weight support paradigms with similar modalities (same intervention time point, speed, with body weight support as needed, and quadrupedal running) but with modified daily intervention time (30 min/d and 60 min/d) and total duration (6 weeks and 12 weeks) were performed [39,40]. The results from this work might suggest that a longer daily duration is required for this type of intervention to be effective, with the total amount of movement as a critical element. In addition, the more severe injury (T11 moderate contusion versus T10 severe contusion) might also contribute to the less effective result. There was research examining the influence of the total amount of movement by comparing results following treadmill training and free-to-access wheel running. Interestingly, the free-access wheel, which allowed unlimited running times during the day, was more effective than the treadmill training (provided limited running times) at ameliorating SCI-NP. Additionally, voluntary wheel running mice demonstrated stronger proprioceptive VGluT1 immunoreactivity than treadmill-trained animals, suggesting a greater activation of proprioceptive afferents [26]. In addition, it was recently reported that participation in voluntary wheel running significantly improved somatosensory evoked potentials at 4 weeks post-SCI [28]. These results suggested that ABI may ameliorate SCI-induced disturbances in proprioception.

There are currently relatively few independent studies that directly compare various ABI modalities. The conclusions presented here are based on comparisons from various investigations, with additional studies necessary to verify these conclusions.

### 4.4. Other Factors May Contribute to the NP Outcome

Having compiled these studies, other factors that may influence NP relief came to light. For example, insufficient housing conditions can lead to the development of stress and depression in laboratory animals, which may exacerbate SCI-NP symptoms [74]. Because laboratory mice/rats are social animals and are highly motivated to interact with one another and with their environment, therefore, environmental and social factors are also likely beneficial to their recovery from experimental interventions or spontaneous diseases [74,75]. Individual housing of laboratory mice/rats may increase vulnerability to surgical stress and interfere with post-surgical recovery [75,76]. Some of the included studies did not detail the housing conditions of the trained animals, making it difficult to obtain information from them [15,19,26,28,69,74]. However, it was found that those experimental animals that were pair- or group-housed post-operatively, with ABI, had positive results for NP remission [15,16,18,19,20,21,22,23,25,28]. Interestingly, 3 of 19 studies mentioned that they housed male animals individually (possibly due to post-surgical males being more likely to fight with each other when group-housed) [16,52,53]. While two of these studies failed to achieve effective NP relief after regular ABI and even presented aggravated mechanical allodynia [14,40], it is unclear if this was due to the solitary housing or the ABI type and parameters used. Given that solitary housing can exacerbate SCI-NP symptoms, it should be considered in future studies to diminish external risk factors.

Sometimes animals are not motivated to participate in ABIs. However, using some unpleasant stimulation (e.g., electric shock) to encourage animals to continue ABI may cause anxiety and induce NP-like behavior [77,78]. Giving rewards to the animal during ABI, such as a food reward [13], may not only enhance the motivation of the animals but may also cause a bias in assessments. Only one study (that reported no impact on NP alleviation) mentioned that their intervention apparatus equipped with electric shocks could be used to stimulate animals to keep walking on the running belt. However, it remains unclear if it was applied [37]. One study with three ABI types used either sugared water or cereal rewards [13]. Either stimulation strategy should not be encouraged for instilling motivation in experimental animals, as it may bias the results. Therefore, one may consider replacements with other practices, such as increased familiar handling and habituation times, to motivate animals in completing the tasks.

## 5. Mechanisms of ABI to Ameliorate SCI-Induced NP

This section will expand on the third section of this article (Section 3. Mechanisms of SCI-Induced NP), which outlined the probable mechanisms or pathways by which SCI leads to NP and will collect evidence on how ABI acts on known mechanisms to alleviate NP.

### 5.1. ABI-Induced Modulations of Inflammatory and Neurotrophic Mediators

**Figure 1:**
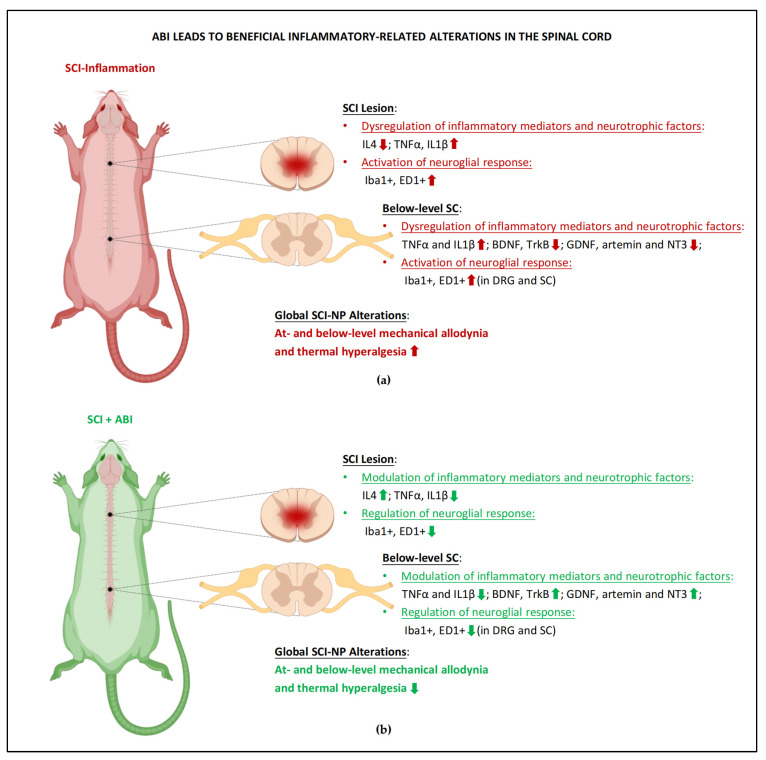
**ABI leads to beneficial inflammatory-related alterations in the spinal cord.** (**a**) Overview of the inflammatory-related alterations in the spinal cord segments of rodents after SCI. Inflammatory cytokines and glial responses increase throughout the at-level lesion site, below-lesion spinal cords, and DRG during various pain states, which is associated with the development of mechanical and thermal hypersensitivity in different body regions that correspond to the affected spinal level. (**b**) Anti-inflammatory signaling was found in the multiple segments of the spinal cord in SCI+ABI animals. ABI-induced anti-inflammatory signals may reduce pro-inflammatory cytokines; while increasing anti-inflammatory cytokines and neurotrophic factor ligand, restoring injury-induced decreases of spinal BDNF levels, leading to reduced mechanical and thermal hypersensitivity normally associated with inflammation. The regulation of extensive neuroglial response also benefits pain relief. The abbreviations that appeared above were explained in the main text. The figure was created with BioRender.com.

Inflammatory responses are persistently increased after SCI (Figure 1); however, an either acute or chronic intervention was successful in stimulating an anti-inflammatory response by suppressing pro-inflammatory mediators TNFα and IL1β and activating anti-inflammatory mechanisms by upregulating the expression of IL4 in the pain pathway for a long period following SCI [23,25]. In addition, ABI was able to decrease the activation of immune cells, such as ED1-expressing macrophages, in at- and below-lesion spinal levels and DRG, by which ABI was reported to ameliorate below-level mechanical allodynia [22]. Furthermore, several interventions can provide analgesic functions by reducing Iba1-expressing microglial activity in the spinal cord dorsal horn below the level of SCI [22,28].

Neurotrophic factors were also regulated by several activity-based paradigms. BDNF, NT-3, and synapsin I were found to be upregulated in different spinal levels with a gradual improvement in below-level NP symptoms [13]. More interestingly, ABI can promote the restoration of GABAergic inhibitory functions in the spinal cord dorsal horn by activating the BDNF/TrkB signaling pathway [19,23,26,27]. On the other hand, the levels of GDNF and artemin expression were normalized in the spinal cord and DRG below lesion level by ABI, along with significant relief of below-level mechanical allodynia and thermal hyperalgesia [15].

### 5.2. Remodeling of Spinal Cord Gray Matter Neural Circuitry

**Figure 2:**
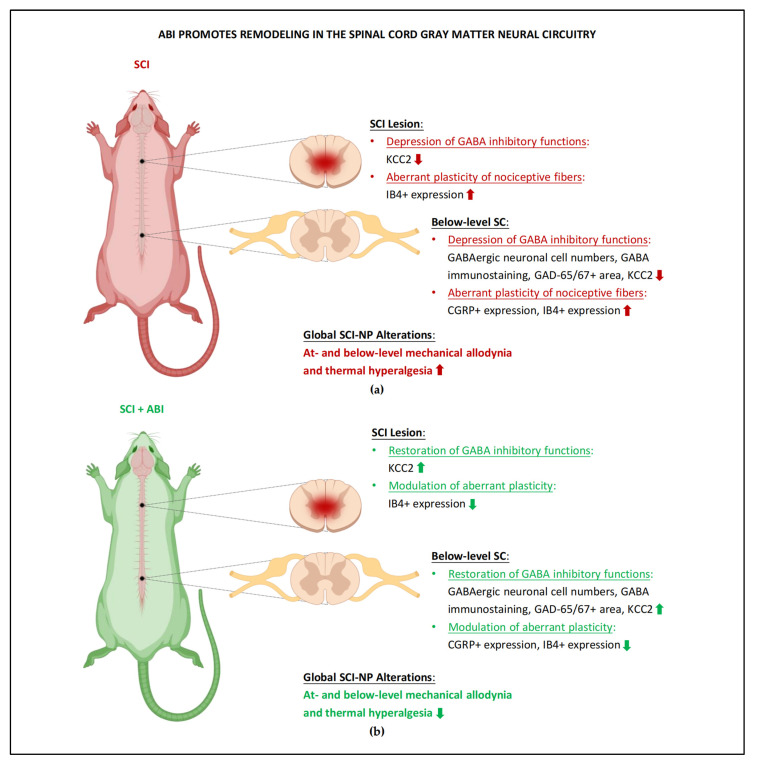
**ABI promotes remodeling in the spinal cord gray matter circuitry.** (**a**) Overview of the maladaptive plasticity in the spinal cord neural circuitry of SCI-NP rodents. GABA inhibition was depressed throughout the lesion site and below-level spinal cords during numerous pain states, and aberrant plasticity of C-/Aδ-fibers was observed in below-level segments, which were associated with the development of mechanical and thermal hypersensitivity. (**b**) Restoration of GABA inhibitory functions and modulation of aberrant plasticity was detected in the spinal cord gray matter neural circuitry of SCI+ABI animals. Reduced CGRP- and IB4-labeling density/expression in the dorsal horn of the lumbar spinal cord demonstrates that aberrant plasticity of nociceptive fibers after SCI can be modulated by ABI of the spinal cord neural circuitry below the lesion, leading to reduced mechanical and thermal hypersensitivity. The abbreviations that appeared above were explained in the main text. The figure was created with BioRender.com.

ABI seems to reduce below-level SCI-NP by facilitating the restoration of the physiological balance of endogenous GABAergic inhibition of the spinal cord dorsal horn pain pathway and modulating maladaptive plasticity of spinal cord gray matter neural circuitry (Figure 2). Such interventions can increase/normalize the expression of BDNF and promote recovery of GABAergic inhibition. In particular, ABI can influence KCC2 levels [23,25] and GAD-65/67 synthesis [21] in distal spinal levels via BDNF/TrkB signaling, as evidenced by the fact that suppressing the pathway reduced the expression of all three markers (KCC2, GAD-65, and GAD-67), as well as the appearance of below-level NP symptoms [17,24]. Several interventions have been proven to decrease excitatory mechanisms, including regulating the function of the perineuronal net (PNN), and the reduction of its expression may reveal the hyperexcitatory state of the spinal cord gray matter neural circuitry below the injury site. This beneficial modulation was supported by the normalized rate-dependent depression of the H-reflex and reduced thermal hyperalgesia [26].

The aberrant plasticity and expression of peptidergic C-/Aδ- fibers in the superficial dorsal horn following SCI can be modulated by ABI, and the amelioration of below-level NP behaviors coincided with reduced CGRP-labeling density in laminae III-IV of the distal spinal cord [17,19,20,21]. Furthermore, the increased expression of CGRP in lamina I-II of L4-L6 lumbar spinal cord was reduced in rats that underwent ABI [28]. The aberrant sprouting of IB4-expressing fibers in laminae II or III-IV caudal to the lesion site was normalized by ABI and associated with a reduction in below-level NP [15,20]. As a result, those interventions may be able to modulate the hyperexcitable phenomenon in the spinal cord gray matter neural circuitry and provide effective analgesia to below-level NP.

## 6. Conclusions

In summary, our current review offers several distinct perspectives on ABI attenuating SCI-NP. Weight-bearing rhythmic paradigms seem to be effective whether started early or delayed or applied short- or long-term but may not carry over for long once halted. Multiple activity-based paradigms worked through inflammatory signaling reduction, restoration of local inhibitory mechanisms of dorsal horn neurons, and modulation of maladaptive plasticity in the spinal cord gray matter circuitry. However, due to the temporary scarcity of studies on this particular scope, the reproducibility of mechanistic findings requires further consideration. Therefore, independent validation of single findings is strongly recommended to increase the certainty of the work.

We also suggest further improving preclinical research by exploring aspects that have been ignored thus far, such as combining more emotional components of NP-related assessments and exploring the characteristics of SCI-NP together with the effects of ABI in both sexes and various experimental models. These represent relevant aspects towards clinical translation. Already, we provide here the essential analgesic elements of ABI to be considered when developing an individualized therapeutic method in the clinic. According to patients’ injury severity and functional recovery over time, the intervention timepoint (early or delayed) can be judged; continuous and regular weight-bearing rhythmic ABI (e.g., treadmill training, task-oriented walking, etc.) can be employed depending on the recovery extent, which may be an effective form of ABI for alleviating SCI-NP based on the preclinical findings. A further refined ABI treatment may provide improved results. Moreover, studies evaluating combinatorial treatment, such as ABI combined with first-line treatments (e.g., medication interventions), with the examination of mechanistic aspects, including pain mediators and structural alterations, are necessary.

## Figures and Tables

**Table 1:**
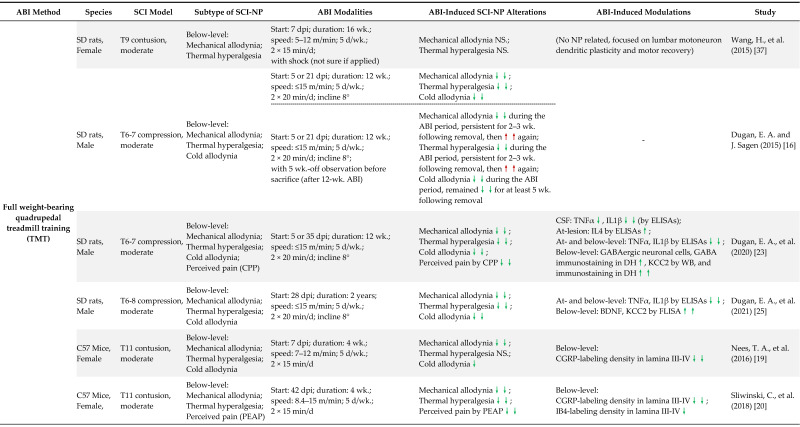

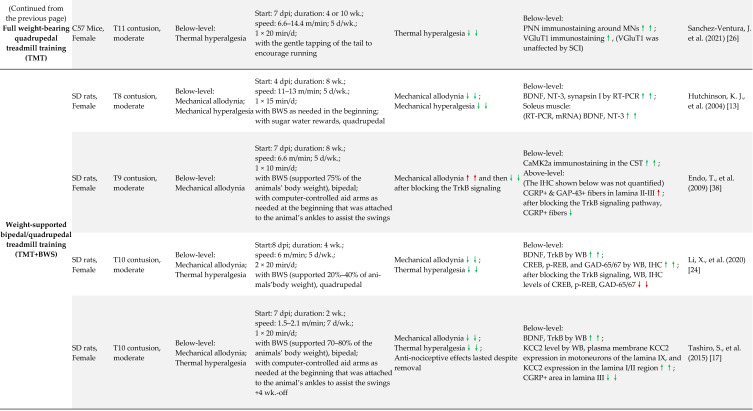

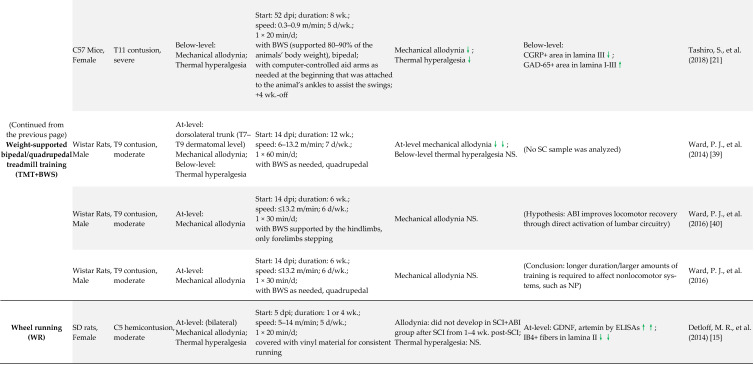

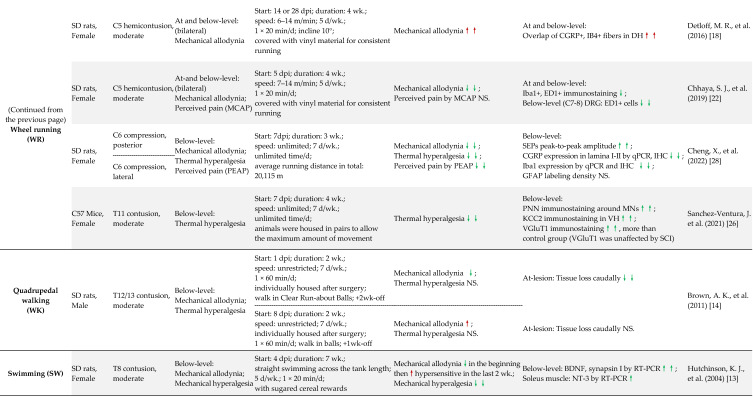

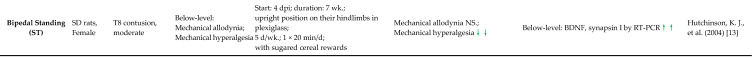
Preclinical studies examining the effect of ABI on spinal cord injury-induced neuropathic pain (SCI-NP).

**RED** signifies the behavioral/mechanistic development of NP; **GREEN** signifies the behavioral/mechanistic relief of NP. **↑↑/↓↓**: Results showed statistical significance in comparison to controls.; **↑/↓**: behavioral/mechanistic results did not reach statistical significance but deemed by the authors as potentially worthy of further examination; **NS**: No statistical significance. All abbreviations used in tables are explained in the main text. The above legends apply to all tables.

**Table 2:**
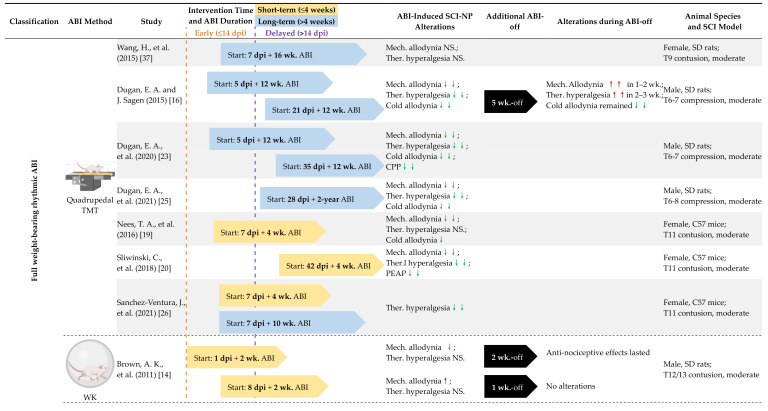

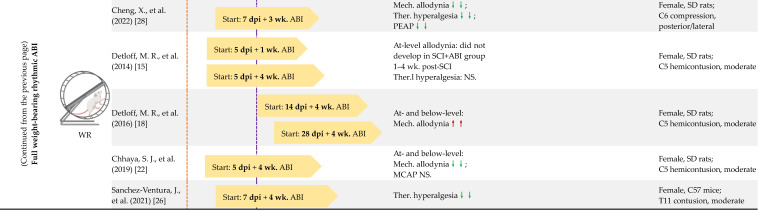

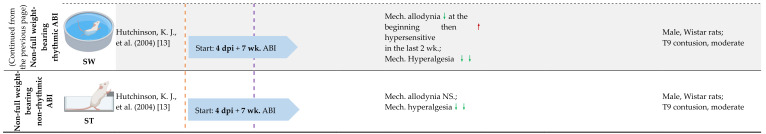

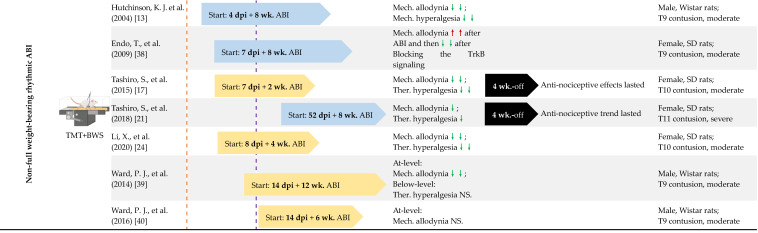

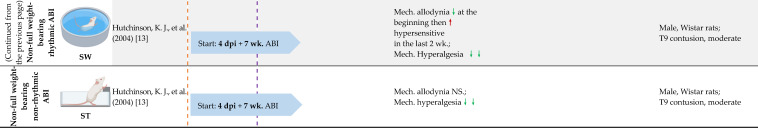
Several factors affecting the efficacy of ABI (time of intervention, duration, and weight bearing).

Pain symptoms (allodynia/hyperalgesia, etc.) that were not specifically indicated in the **ABI-induced SCI-NP Alterations** column in this table are referring to below-level NP symptoms. For the interpretation of the symbols, please refer to the legend in Table 1. The **BLUE/YELLOW** arrows in the table are not drawn to scale but do represent relative differences in the duration of the ABI. **Abbreviations: ABI**: Activity-Based Intervention (s); **BWS**: Body Weight Support; **CPP**: Conditioned Place Preference; **DH**: (Spinal Cord) Dorsal Horn; **Mech.**: Mechanical; **MCAP**: Mechanical Conflict-Avoidance Paradigm; **MN(s)**: Motoneuron(s), **PEAP**: Place Escape/Avoidance Paradigm; **PNN**: Perineuronal Net(s); **SW**: Swimming; **Ther.**: Thermal; **TMT**: Treadmill Training; **VH**: (Spinal Cord) Ventral Horn, **wk.**: week (s); **WK**: Walking; **WR**: Wheel Running.

**Table 3:**
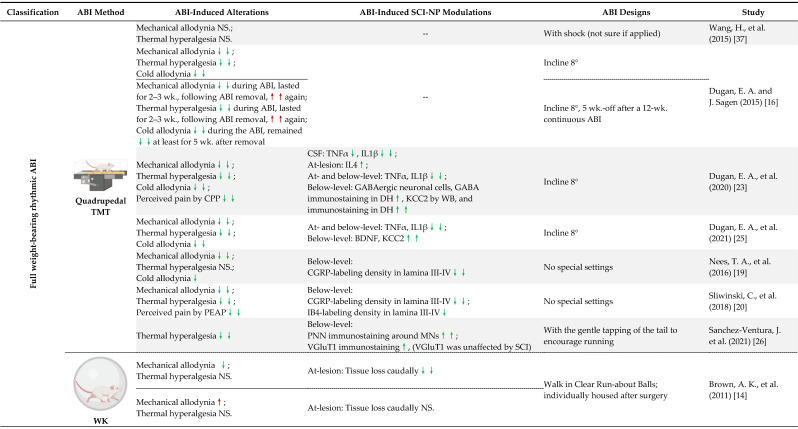

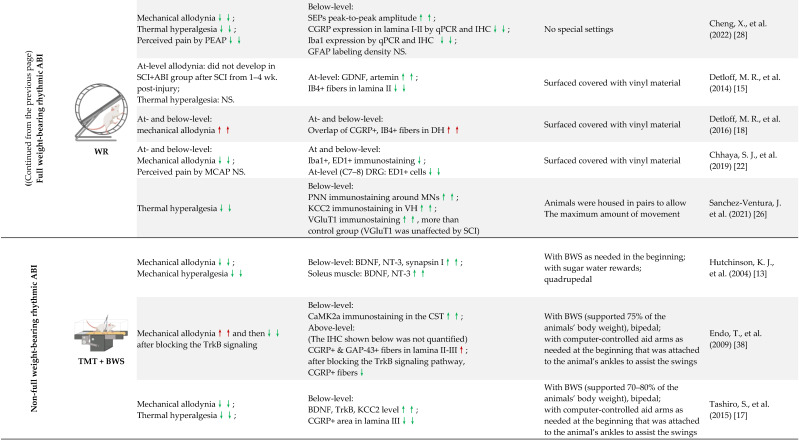

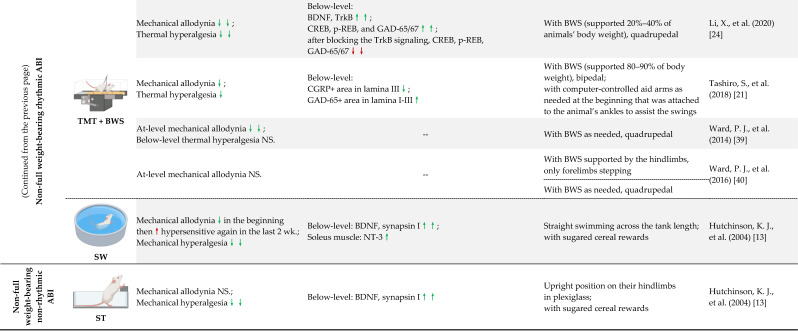
Full-Weight Bearing vs. Non-Full Weight-Bearing Rhythmic ABI.

Pain symptoms (allodynia/hyperalgesia, etc.) that were not specifically indicated in the **ABI-induced SCI-NP Alterations** column in this table are referring to below-level NP symptoms. For the interpretation of the symbols, please refer to the legend in Table 1. **Abbreviations: ABI**: Activity-Based Intervention (s), **BWS**: Body Weight Support; **CPP**: Conditioned Place Preference; **DH**: (Spinal Cord) Dorsal Horn; **MCAP**: Mechanical Conflict-Avoidance Paradigm; **MN (s)**: Motoneuron (s); **PEAP**: Place Escape/Avoidance Paradigm; **PNN**: Perineuronal Net (s); **SW**: Swimming; **TMT**: Treadmill Training; **VH**: (Spinal Cord) Ventral Horn; **wk.**: week (s); **WK**: Walking; **WR**: Wheel Running.

## Data Availability

Not applicable.

## Abbreviations

The abbreviations that appear in the present review (including tables and figures) are listed as follows:

ABI	Activity-Based Intervention(s);
BDNF	Brain-Derived Neurotrophic Factor;
BWS	Body Weight Support;
CGRP	Calcitonin Gene-Related Peptide;
CPP	Conditioned Place Preference;
CSF	Cerebrospinal Fluid;
DH	(Spinal Cord) Dorsal Horn;
dpi	Day(s) Post Injury;
DRG	Dorsal Root Ganglia;
GAD-65/67	Glutamic Acid Decarboxylase-65/67;
GDNF	Glial Cell-Derived Neurotrophic Factor;
GFL	GDNF Family of Ligands;
IB4	Isolectin B4;
KCC2	The Neuron-Specific K^+^ Cl^−^ Cotransporter;
MCAP	Mechanical Conflict-Avoidance Paradigm;
Mech.	Mechanical;
MN (s)	Motoneuron(s);
NP	Neuropathic Pain;
PEAP	Place Escape/Avoidance Paradigm;
PNN	Perineuronal Net(s);
SCI	Spinal Cord Injury;
SCI-NP	Spinal Cord Injury-Induced Neuropathic Pain;
SD rats	Sprague–Dawley rats;
ST	Standing;
SW	Swimming;
Ther.	Thermal;
TMT	Treadmill Training;
TrkB	Tropomyosin-Related Kinase B;
VH	(Spinal Cord) Ventral Horn;
WB	*West.* Blot;
wk.	week(s)
WK	Walking;
WR	Wheel Running
